# A Neuroprotective Dose of Isatin Causes Multilevel Changes Involving the Brain Proteome: Prospects for Further Research

**DOI:** 10.3390/ijms21114187

**Published:** 2020-06-11

**Authors:** Alexei Medvedev, Arthur Kopylov, Olga Buneeva, Leonid Kurbatov, Olga Tikhonova, Alexis Ivanov, Victor Zgoda

**Affiliations:** Department of Proteomic Research and Mass Spectrometry, Institute of Biomedical Chemistry, 10 Pogodinskaya Street, 119121 Moscow, Russia; a.t.kopylov@gmail.com (A.K.); olbuneeva@gmail.com (O.B.); kurbatovl@mail.ru (L.K.); tiolika@gmail.com (O.T.); professor-ivanov@yandex.ru (A.I.); victor.zgoda@gmail.com (V.Z.)

**Keywords:** neuroprotector isatin, molecular targets, differentially expressed proteins, proteome analysis, isatin-binding proteins, interactome

## Abstract

Isatin (indole-2,3-dione) is an endogenous regulator, exhibiting a wide range of biological and pharmacological activities. At doses of 100 mg/kg and above, isatin is neuroprotective in different experimental models of neurodegeneration. Good evidence exists that its effects are realized via interaction with numerous isatin-binding proteins identified in the brain and peripheral tissues studied. In this study, we investigated the effect of a single dose administration of isatin to mice (100 mg/kg, 24 h) on differentially expressed proteins and a profile of the isatin-binding proteins in brain hemispheres. Isatin administration to mice caused downregulation of 31 proteins. However, these changes cannot be attributed to altered expression of corresponding genes. Although at this time point isatin influenced the expression of more than 850 genes in brain hemispheres (including 433 upregulated and 418 downregulated genes), none of them could account for the changes in the differentially expressed proteins. Comparative proteomic analysis of brain isatin-binding proteins of control and isatin-treated mice revealed representative groups of proteins sensitive to isatin administration. Control-specific proteins (n = 55) represent specific targets that interact directly with isatin. Appearance of brain isatin-binding proteins specific to isatin-treated mice (n = 94) may be attributed to the formation of new clusters of protein–protein interactions and/or novel binding sites induced by a high concentration of this regulator (ligand-induced binding sites). Thus, isatin administration produces multiple effects in the brain, which include changes in gene expression and also profiles of isatin-binding proteins and their interactomes. Further studies are needed for deeper insight into the mechanisms of the multilevel changes in the brain proteome induced by isatin. In the context of the neuroprotective action, these changes may be aimed at interruption of pathological links that begin to form after initiation of pathological processes.

## 1. Introduction

Isatin (indole-2,3-dione) is an endogenous oxidized indole found in the brain, peripheral tissues, and biological body fluids of humans and animals [1,2,3,4,5]. The interest of researchers in this “talented molecule” [6] is determined by various regulatory effects described in the literature (see [1,7] for review) and the role of isatin as a core structure in numerous synthetic isatin-based compounds exhibiting different pharmacological activities [7,8,9]. Numerous isatin analogues have been synthesized and tested as anticonvulsants [7,10], antibacterial [7,11], antiviral [7,12,13,14], anticancer agents [8], and inhibitors of apoptosis [15,16]. Sunitinib (marketed by Pfizer as Sutent), a 5-fluoro-3-substituted isatin derivative, was approved by the FDA (U.S. Food and Drug Administration) for treatment of advanced renal cell carcinoma [17].

Isatin has also been found in mammalian brain, peripheral tissues, and body fluids [18,19,20,21,22,23], and there is experimental evidence for its endogenous origin [24]. Various types of stress have a significant impact on isatin levels in the brain, serum, urine, and examined tissues. In rats exposed to immobilization/audiogenic stress, the isatin levels in the brain, heart, and serum were 2–4-fold higher than in control animals [21]. Cold-stressed rats (for 2 h at 4°C) exhibited a significantly higher (2–3-fold) isatin content in the daily (24 h) urine [22]. Food deprivation for three days (with free access to water) caused even a more pronounced (~5-fold) increase in isatin in the daily urine [22]. Administration of the proconvulsant, pentylenetetrazole, increased (~1.5-fold) the brain isatin content [23].

Exogenous isatin readily crosses the blood brain barrier; for example, isatin injection to rats at a dose of 50–100 mg/kg increased the level of brain isatin up to 9 µg/g [23]. Isatin administered in vivo produced various (dose-dependent) physiological/pharmacological effects. Low doses of isatin (15–20 mg/kg) were anxiogenic in open field and elevated plus maze tests in albino mice [25,26] and in social interaction test in rats, while the locomotor activity of these rats remained unchanged [25]. In contrast to mice, rats were insensitive to the anxiogenic doses of isatin in the open field and forced swim tests [27], and higher doses of isatin (80–160 mg/kg) caused sedation manifested as the reduced distance in the open field and increased immobility in the forced swim test [27]. The higher doses of isatin (from 60 mg/kg to 200 mg/kg) produced an anticonvulsive effect evaluated in different models including audiogenic seizures in rats [28,29] and pentylenetetrazole administration [30].

In rats with the 6-hydroxydopamine model of Parkinsonism, isatin (100 mg/kg) inhibited rotations induced by apomorphine [31]. In the context of other experimental models of Parkinson’s disease, administration of isatin (100 mg/kg) also decreased locomotor impairments in rats with Parkinsonism induced by Japanese encephalitis virus [32,33] and in mice with Parkinsonism induced by the neurotoxin MPTP (1-methyl-4-phenyl-1 2 3 6-tetrahydropyridine) [34,35]. Isatin administration (100 mg/kg) also influenced the profile of ubiquitinated mitochondrial proteins in the mouse brain. These changes were observed 2 h after isatin administration [35].

Biological activities of isatin involve isatin-binding proteins, identified during proteomic profiling of brain preparations of mice and rats [1,36,37,38]. Interestingly, many of these proteins are actively investigated in the context of neurodegenerative diseases such as Parkinson’s disease and Alzheimer’s’ disease [37,39,40]. In vitro physiological concentrations of isatin (1–10 μM) inhibit monoamine oxidase B (*MAO B*) and natriuretic peptide receptor coupled guanylate cyclases [1,5]; higher (neuroprotective) concentrations (50–400 μM) inhibit non-glycolytic activity of glyceraldehyde-3-phopshate dehydrogenase [39], induce apoptosis of various (including malignant) cells, and affect gene expression associated with apoptosis [1,41,42,43,44,45,46]. Some authors even believe that due to its cytotoxic and antiproliferative activities, isatin should be considered a good candidate as a pharmacologically attractive chemotherapeutic substance [47].

It should be noted that after administration of the neuroprotective doses of isatin (50–100 mg/kg), its concentration in the brain may exceed 70 µM [1,4]. Our recent study has shown that treatment of mice with a neuroprotective dose of isatin significantly influenced the proteomic profile of brain isatin-binding proteins [48]. The detected changes in the profile of isatin-binding proteins are consistent with the accumulation of administered isatin in the brain and its binding to target proteins, thus preventing subsequent protein binding to the affinity sorbent containing an isatin analogue as the affinity ligand [48]. Earlier, we also demonstrated that within a slightly different scheme of the animal experiment, pretreatment of rats with a neuroprotector dose of isatin prevented brain *MAO B* against irreversible inhibition by the mechanism-based inhibitor phenelzine [49]. Cell culture studies revealed that incubation with high concentrations of isatin (100 µM and above) influenced the expression of some genes [43,44,45,50,51].

Thus, multiple biological/pharmacological effects of isatin may be attributed to both isatin interaction with particular protein targets (isatin-binding proteins) and also regulation of isatin-responsive genes. However, proteomic profiling of differentially expressed proteins and their possible association with altered expression of corresponding genes have never been investigated after isatin administration in vivo. Moreover, possibility of the regulation of gene expression in the brain of animals by administration of a single dose of isatin has not been investigated at all; certain evidence only exists that chronic treatment of animals with a low dose of isatin influenced the expression of some selectively studied genes in the brain [52].

Thus, the aim of this work was to investigate the effect of administration of isatin (100 mg/kg) to mice one day before analysis on the following: (i) differently expressed proteins in brain hemispheres; (ii) their possible association with altered gene expression; and (iii) profile of isatin-binding proteins in the brain hemispheres. Previous studies have shown that this dose of isatin attenuated manifestations of MPTP-induced Parkinsonism in mice [34,35]. The time interval of 24 h was chosen on the basis of our analysis of literature data on altered expression of genes in cell cultures treated with isatin [1]. Such a time interval is often used for the drug-induced preconditioning of the brain [53].

## 2. Results

### 2.1. The Effect of Isatin Administration to Mice on Differently Expressed Proteins in Brain Hemispheres

Isatin administration to mice caused downregulation of 31 proteins (Table 1). The most pronounced changes were found in the case of calcium/calmodulin-dependent protein kinase type IV (about 11-fold), fructose-1,6-bisphosphatase 1 (more than 8-fold), serine protease inhibitor A3K (more than 4-fold), nucleolar protein 3 (almost 4-fold), and neurobeachin (more than 3-fold). These and other proteins listed in Table 1 participate in processes involved in cell signaling, regulation of cell death and proliferation, and also in protein synthesis. In this context, it is particularly interesting to note that fructose-1,6-bisphosphatase 1 (FBP1), a classical glycolytic enzyme, may block the transcriptional activity of the hypoxia-inducible factor (HIF-1α) and prevent activation of the RAS/RAF/MEK/ERK pathway (see [54] for review).

**Figure 1:**
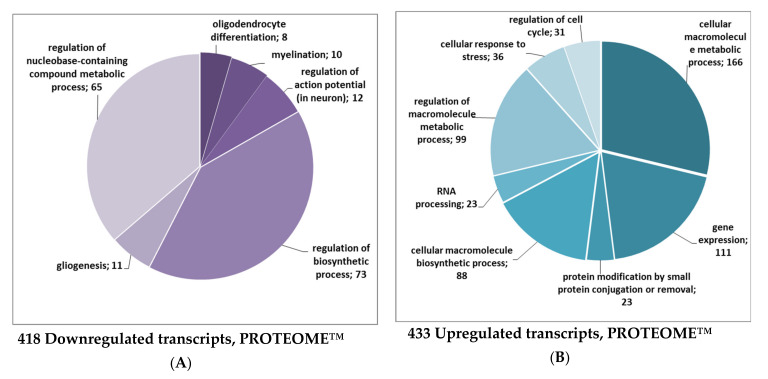
Functional annotation of transcriptome data in terms of Gene Ontology (GO) biological processes. Color intensity reflects the significance of enrichment: darker colors indicate higher statistical significance. The biological process in terms of GO supported the prevalence of downregulated transcripts related to the regulation and development of neurons including those guiding gliogenesis and myelination (**A**). The upregulated transcripts are related to processes involved in the response to DNA damage and consequent regulation of cell death as well as negative regulation of common cellular processes in the population of upregulated transcripts (**B**). Since each gene may be involved in several biological processes, the number of up- and downregulated transcripts does not match the sum of biological processes shown in the Figure. Other explanations are given in the text.

However, the changes in differentially expressed proteins cannot be attributed to altered expression of corresponding genes. Although at this time point isatin influenced the expression of more than 850 genes in brain hemispheres (including 433 upregulated and 418 downregulated genes), none of them could account for the changes in the differentially expressed proteins listed in Table 1. In total, transcripts of 19,903 genes (including 19,052 non-changed transcripts) were detected. Table 2 shows the top 20 upregulated and downregulated genes. Most genes listed in this table are involved in processes related to cell proliferation and signaling. This trend is also supported by Gene Ontology classification terms for all differentially expressed genes where several groups for different biological process regulations and gene expressions were found (Figure 1).

Proteomic analysis revealed 1058 proteins corresponding to detected transcripts. However, correlation between the expression of all identified proteins and their transcripts was very low (R = 0.14) and did not reach the level of statistical significance. Combined transcriptomic and proteomic analyses revealed only two matched transcript/protein coding genes. However, in both cases these included an increase in the transcript level and a decrease in the protein level: (i) Q9JLV5, cullin 3: a 3.2-fold increase in the transcript level and a 2.7-fold decrease in the protein level; (ii) Q61029, thymopoietin: a 5.9-fold increase in the transcript level and a 2.1-fold decrease in the protein level (Table 1).

Using the PROTEOME™ database for functional classification, we found downregulated transcripts important for nervous system development (n = 8, GO:0048709 oligodendrocyte differentiation, adjusted *p* value = 0.022; n = 10, GO:0042552 myelination, adjusted *p* value = 0.027; n = 11, GO:0042063 gliogenesis, adjusted *p* value = 0.043) and function (n = 12, GO:0001508 regulation of action potential in neuron, adjusted *p* value = 0.026; Figure 1A). These included the following transcripts: *Fa2h*, *Gjb1*, *Gpr17*, *Qk*, *Rcor2*, *Sox10*, *Traf4*, *Yy1*, *Dok4*, *Gal3st1*, *Gmfb*, *Kcnj9,* and *Notch2*. The mean log-fold decrease for 418 downregulated transcripts (Figure 1A) was FC= –0.87 and ranged between –2.796 and –0.501 with a *p* value < 0.001).

The most upregulated transcripts (Figure 1B) were related to biological processes involved in the cell metabolic process (n = 166, GO:0044260, adjusted *p* value = 1.44 e-12) and its regulation (n = 99, GO:0060255, adjusted *p* value = 5.35e-06), gene expression (n = 111, GO:0010467, adjusted *p* value = 1.68e-06), cellular macromolecule biosynthetic process (n = 88, GO:0034645, adjusted *p* value = 2.98 e-06), cellular response to stress (n = 36, GO:0033554, adjusted *p* value = 1.04e-05), regulation of cell cycle (n = 31, GO:0010941, adjusted *p* value = 8.14e-05), and RNA processing (n = 23, GO:0006396, adjusted *p* value = 2.98e-06). The mean log-fold increase for all upregulated transcripts (n = 433) was FC = 0.83 and ranged between 0.504 and 3.132 at *p* < 0.001. Remarkably, some GO groups related to metabolic and biosynthetic processes (e.g., response to DNA damage stimulus (n = 45, GO:0006974, adjusted *p* value = 1.84e-06), regulation of cell death (n = 82, GO:0010941, adjusted *p* value = 7.24e-06), and the group responsible for nucleic acid metabolic process (n =193, GO:0090304, adjusted *p* value = 2.59e-12) were similar for both groups.

Expectedly, both up- and downregulated transcripts were localized in the nucleus (n = 296, GO:0005634, adjusted *p* value = 2.19e-15) or were components of the nucleoplasm (n = 79, GO:0005654, adjusted *p* value = 2.70e-10). The prevalence of downregulated transcripts in the cytosol (n = 209, GO:0005737, adjusted *p* value = 1.10e-07) or in macromolecular complexes (n = 271, GO:0032991, *p* = 3.22e-05) was higher compared to the upregulated elements (n = 154, adjusted *p* value = 1.63e-07, and n = 169, adjusted *p* value = 3.19e-05, respectively) (see Supplementary Materials S1).

Results of our study suggest that at 24 h after administration, the neuroprotective dose of isatin downregulated some brain-specific processes (and corresponding genes) and upregulated the expression of genes involved in the stress response.

### 2.2. Isatin-Binding Proteins

Besides isatin-binding proteins common in brain hemispheres of control and isatin-treated mice (Supplementary Materials S2), there were isatin-binding proteins specific to each group of animals (Supplementary Materials S3). Control-specific isatin-binding proteins (n = 55) represent direct targets of isatin, as their interaction with the immobilized isatin analogue was influenced (inhibited) by isatin administration to animals. In this context, the accumulation of exogenously administered isatin in the brain and its interaction with target proteins in vivo (i.e., before brain tissue processing for subsequent analysis) had a significant impact on the proteomic profiling. Such homologous competition for particular targets in the brain was used earlier for evaluation of the specific binding of [^3^H]isatin in various brain structures [36]. In addition, according to our previous calculations made on the basis of the brain isatin content and the brain tissue water content [1,4], after administration of 50–100 mg/kg isatin to animals, its concentrations in the brain could be as high as 70 µM (provided that all the exogenously administered isatin was evenly distributed in the brain) or even higher (in the case of uneven distribution in the brain tissue). Earlier, it was also found that pretreatment of rats with a dose of isatin 80 mg/kg significantly attenuated irreversible inactivation of brain MAO B by the mechanism-based inhibitor phenelzine [49]. All this supports the mechanism of competitive interactions between isatin accumulated in the brain (and bound to its targets) in vivo and the affinity ligand, 5-aminoisatin-Sepharose, added to concentrated brain lysates in vitro (see Materials and Methods Section).

**Figure 2:**
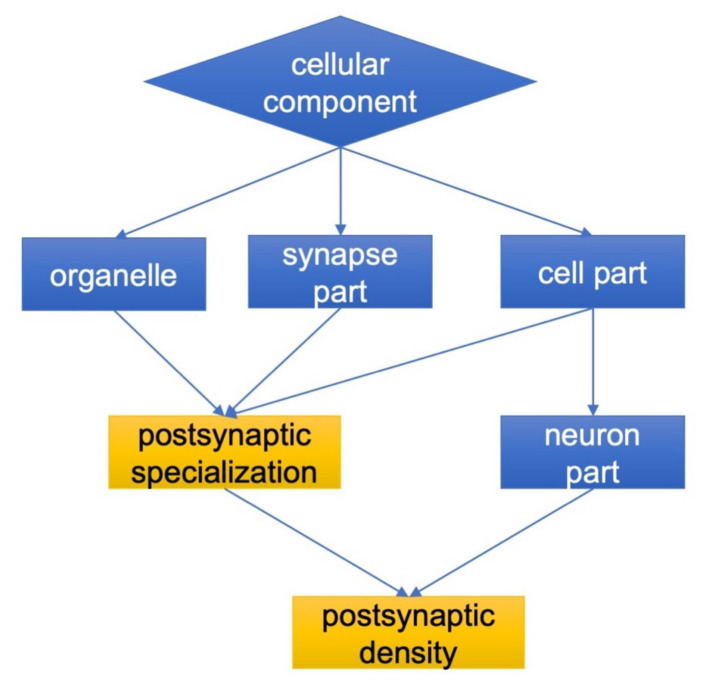
Cellular localization and functional links of brain isatin-binding proteins specific to isatin-treated mice. Details are given in the Materials and Methods Section.

In the case of brain isatin-binding proteomes of isatin-treated mice, there were 94 proteins that were absent in the fraction of brain isatin-binding proteins of control mice. Their appearance may be associated with several possible mechanisms including isatin-induced changes in protein–protein interactions as well as isatin-induced formation of new binding sites (see the Discussion section). However, regardless of the particular mechanism(s), it should be noted that these isatin-binding proteins have particular cellular localization and functional links as evidenced by GO terms (Figure 2, see also Supplementary Materials 3). In contrast to control-specific isatin-binding proteins which were not linked (assigned) to the particular cell compartments, the brain isatin-binding proteins specific to the isatin-treated mice could be well characterized by targeted localization related to neuronal synapses (see Figure 2).

## 3. Discussion

Administration of the neuroprotective dose of isatin (100 mg/kg, s.c.) to mice had a significant impact on both the expression of genes and the profile of isatin-binding proteins.

Results of transcriptome and proteome analyses of differentially expressed genes suggest the lack of concerted changes 24 h after administration of the neuroprotective dose of isatin. In other words, changes in the transcripts of genes have not been translated into corresponding changes of their protein products. In addition, unidirectional changes occurred in genes/proteins involved in opposite processes such as cell proliferation and cell death. This may be attributed to the known phenomenon of uncoupling of transcription and translation when some delay between transcription and translation occurs in the course of dynamic adaptation processes [55]. Since the time interval of 24 h after isatin administration was selected as the earliest period for the detection of altered gene expression, it is possible that concerted changes in the gene expression stages may appear later, after reaching a new steady state of the dependence of the protein level on the respective mRNA concentration [55]. On the other hand, the protein level in the cell is not strictly dependent on the expression of particular genes as it involves other factors, including posttranslational modifications, needed for both biological activity of particular proteins and also for their (targeted) elimination/degradation. These discrepancies clearly need further studies including several time points (i.e., longer intervals after isatin administration) and validation by independent approaches.

**Figure 3:**
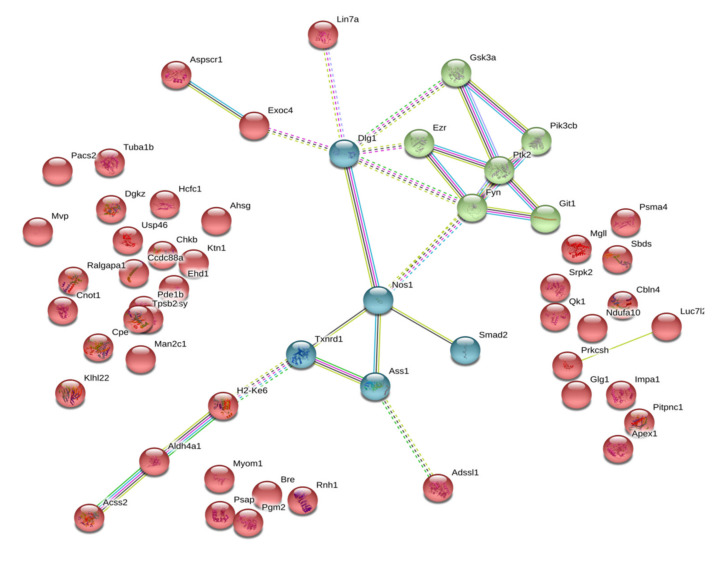
Functional links between control-specific isatin-binding proteins of the mouse brain hemispheres. Line thickness and type (solid or dashed) reflect combined score and evidence suggesting a functional link. Bold lines indicate direct interactions between the identified proteins, annotated in String (v. 11.0 of January 19, 2019). Bold dashed lines show experimentally proven interactions between the identified proteins. Colors indicate different functional clusterization: red color shows lack of functional interactions, green color designates interaction between structural and regulatory proteins, blue color indicates interaction of proteins involved redox-dependent regulation.

Administration of the neuroprotective dose of isatin to mice influenced the profile of brain isatin-binding proteins. Although profiles of brain isatin-binding proteins of control and isatin-treated mice shared significant similarity, pools of specific isatin-binding proteins were recognized in both groups of animals. Figure 3 shows that functionally control-specific isatin-binding proteins mainly exist as independent molecules unrelated to each other: the majority of these isatin-binding proteins (37 of 55) demonstrate rather poor functional interactions, thus suggesting their involvement in distinct, unrelated processes. This is the only axis that links documented interactions between neuronal nitric oxide synthase (*NOS1*), generating nitric oxide from arginine, argininosuccinate synthetase, the enzyme crucial for arginine synthesis, and the antioxidant enzyme thioredoxin reductase 1 (*Txnrd1*), as well as components of the regulatory pathways, which share Disks large homolog 1 (*Dlg1*) as a hub.

*DLG1* is a member of the membrane-associated guanylate kinase (MAGUK) family of proteins, which function as molecular scaffolds. *Dlg1*, also known as synapse-associated protein 97 or *SAP97*, is a scaffolding protein that belongs to the MAGUK family [56]. Being involved in the formation of specific multiprotein complexes, including receptors, ion channels, and signaling proteins, it is implicated in the control of cell polarity. *Dlg1* promotes the arrangement of various assemblies of cell adhesion molecules, receptors and/or ion channels, and other signaling pathway components with cytoskeleton proteins at particular plasma membrane regions [56]. Recently, it was demonstrated that *Dlg1* (SAP97) co-precipitated with neuronal *NOS* (*NOS1*), and this interaction is important for regulatory thiol modification of GluA1 AMPA receptors [57]. Implication in local redox modifications explains the direct interaction between *NOS1* and Thioredoxin reductase 1 (Txnrd1), while *NOS1* and *Smad2* are both involved in TGF-β signaling [58,59].

The other functional cluster containing well-documented interacting proteins includes proteins *GSK3a*, *Pik3cb*, *Ptk2*, *Fyn* (nonreceptor tyrpsine kinase), *Git1* (ARF GTPase-activating protein), and *Ezr*, which interacts with *Dlg1* via its FERM domain [60]. Being one of the ERM proteins (ezrin, radixin, and moesin) forming a highly conserved branch of the FERM domain (four-point-one, ezrin, radixin, moesin) [61], ezrin functions as a membrane-cytoskeleton linker involved in the connections of major cytoskeleton structures to the plasma membrane [62]. It adopts various conformational states [60], which promote ezrin interactions with various partners including different kinases, which can phosphorylate ezrin and other members of this family (radixin and moesin) at the FERM domain [61].

Ezrin forms a functional triagle, including protein tyrosine kinase 2 (*PTK2*), also known as focal adhesion kinase (*FAK*), and *Git1*. Protein tyrosine kinase 2 (*PTK2*) is a non-receptor tyrosine-protein kinase [63]; it directly interacts with Ezrin in connections of major cytoskeleton structures to the plasma membrane [62]. *FAK* (*PTK2*) can directly interact with Fyn, a member of the *Src* family of protein tyrosine kinases (SFKs) in lipid rafts [64]. *GIT1*, GTPase-activating protein for the ADP ribosylation factor family, acts as a scaffold crucial for signaling modules with various kinases, including *PTK2* [65]. Interestingly, *GIT1* may serve as an endothelial nitric-oxide synthase (*eNOS*) interactor, contributing to phosphorylation and activation of this enzyme [65], and as the adaptor for actin regulators and local modulator of *Rac* activity at synapses [66].

Glycogen synthase kinase-3 alpha (*GSK3A*) is a constitutively active protein kinase involved in the hormonal regulation of glucose homeostasis, Wnt signaling and also in the regulation of various transcription factors and microtubules [67]. This enzyme is an important target of the PI3K pathway [68]. Good evidence also exists that PI3K signaling via GSK3 may regulate DNA methylation [69].

**Figure 4:**
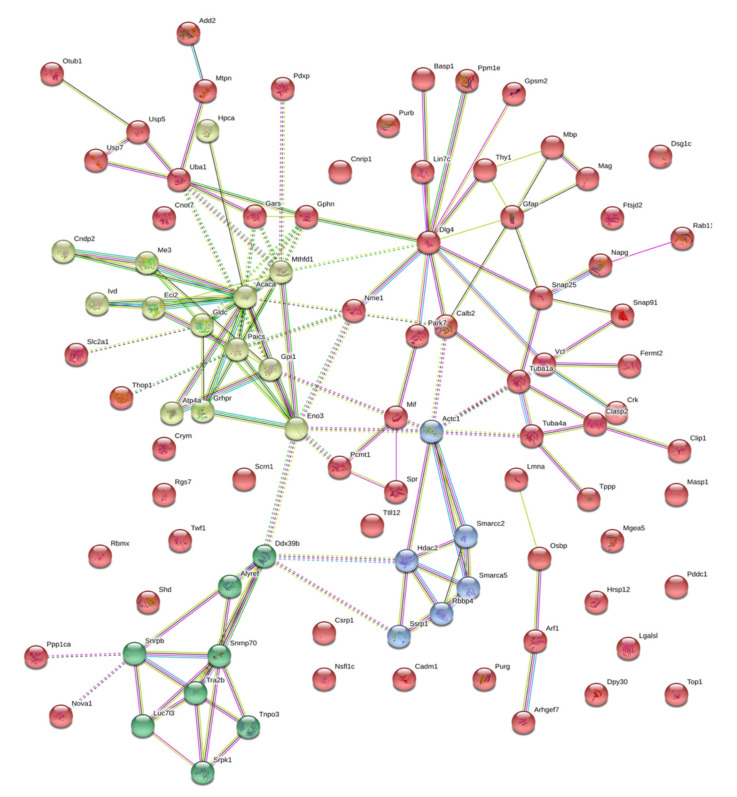
Functional links between isatin-binding proteins specific to the brain hemispheres of isatin-treated mice. Line thickness and type (solid or dashed) reflect combined score and evidence suggesting a functional link. Bold lines indicate direct interactions between the identified proteins, annotated in String (v. 11.0 of January 19, 2019). Bold dashed lines show experimentally proven interactions between the identified proteins. Colors indicate different functional clusterization: red color shows intracellular molecular scaffolds with various intracellular proteins, aquamarine designates proteins involved in regulations of RNA splicing, green color shows enzymes, involved in metabolic processes, blue color shows proteins involved in regulation of neuron-specific proliferation.

In the case of brain isatin-binding proteomes of isatin-treated mice, there were 94 proteins that were absent in the fraction of brain isatin-binding proteins of control mice. Most of them form a deeply integrated protein network in which another member of the membrane-associated guanylate kinase (MAGUK) family of proteins, *DLG4* (known as synapse-associated protein 90, *SAP90*, and the *PSD-95* protein), serves as a hub linking together several clusters of proteins involved in chromatin modification, cytoskeleton formation/rearrangement and intracellular trafficking, metabolic processes, posttranslational modification of proteins, and signaling (Figure 4).

Appearance of these rather large groups of proteins may be attributed to changes in the protein–protein interaction (PPI) networks induced by high isatin concentrations in the brain. In some cases, the appearance of such PPIs may be accompanied by formations of new binding sites.

For example, in *Escherichia coli* cells, temperature- and nucleotide-dependent modulation of the interaction between the central component of translocase (*SecA*) and the integral *SecYEG* membrane protein complex (so called *SecA*–*SecYEG* interaction) is involved in the formation of new binding sites [70]. The other relevant example includes human serum albumin which is able to adopt numerous conformations in dependence on the nature of bound ligand [71].

Platelet glycoprotein IIb/IIIa receptor antagonists can induce conformational changes in the receptor, known as a ligand-induced binding site [72]. In addition, some of brain isatin-binding proteins specific to the group of isatin-treated mice were already identified among the proteins specifically bound to the other isatin analogue (e.g., glial fibrillary acidic protein, *GFAP*) [37]. Although it remains unclear whether these new PPIs result in the formation of tight complexes that bind to the affinity ligand or whether they represent secondary interactions facilitating the isolation of these proteins in the group of isatin-binding proteins [73], there is increasing evidence that isatin may act as a PPI regulator [1]. It should be noted that isatin promoted protein complex formation at least in some model systems, in which individual components demonstrated low isatin-binding activity [74].

The appearance of new functional protein clusters related to the regulation of gene expression-transcription and splicing as well as protein synthesis can account for uncoupling between transcription and translation observed after administration of the neuroprotector dose of isatin: isatin-induced changes in the expressions of genes did not result in corresponding changes of their protein products (Table 1 and Table 2).

In this context, the *Tra2b* protein (the transformer 2 beta homolog) is particularly interesting as it is considered one of the most important splicing factors involved in RNA processing needed for both normal (e.g., brain development) and pathological (e.g., cancer) cell proliferation [75,76]. The RNA binding protein *Rbmx*, also known as heterogenous nuclear ribonucleoprotein G (*hnRNPG*), also functions as an important protein, being involved in the regulation of gene expression and alternative splicing. Using its so-called low-complexity region rather than the canonical RNA-binding domain, *hnRNPG* recognizes and binds a motif exposed by RNA m6A modification (N6-methyladenosine) [77]. Another small nuclear ribonucleoprotein-associated protein B (*SNRPB*) has a significant impact on the expression and processing of spliceosome components [78].

*Gars* (glycyl-tRNA synthetase) catalyzes the reaction of glycine ligation to its cognate tRNA [79,80] needed for global protein synthesis. In humans, impaired functioning of Gars results in the development of hereditary axonal neuropathy known as Charcot–Marie–Tooth disease (CMT) [79,80]. In addition, it is suggested that *GARS* may exhibit axon-specific functions unrelated to tRNA charging [80].

These selected examples suggest that brain isatin-binding proteins specific to isatin-treated animals may directly affect both transcription and translation processes. Taking into consideration that all known effects of isatin on catalytically active protein targets result in the decrease of their activities [1], it is reasonable to suggest that the appearance of new clusters of interactomes in the brain of isatin-treated mice causes a coordinated decrease of several groups of biological processes. All these considerations suggest that the presence of functionally related proteins in the same fraction was not accidental.

Our previous proteomic studies have shown that in mice exposed to a shorter period of time after administration of isatin (100 mg/kg; 90–120 min), there was a significant decrease in the repertoire of brain proteins specifically bound to the Rpn-10 subunit of proteasomes [24] and a number of proteins associated with the brain mitochondrial fraction [25].

This study has shown that 24 h after the administration of the neuroprotective dose of isatin, the brain hemispheres responded by downregulation of 31 proteins, altered expression of genes, uncoupling between transcription and translation processes, as well as by changes in the profiles of isatin-binding proteins. The changes in the profiles of isatin-binding proteins included the disappearance of control-specific individual proteins from the isatin-binding pattern due to loading with administered isatin (and homologous competition with an isatin analogue as the affinity sorbent) and the formation of new clusters of interacting proteins specific to the isatin-treated brain.

These effects are aimed at interruption of pathological links that begin to form after initiation of pathological processes such as MPTP-induced parkinsonism [34,35]. It is possible that some of the recognized proteins and genes sensitive to the neuroprotective dose of isatin may be used as potential targets for numerous analogues synthesized in many laboratories worldwide [81,82,83].

## 4. Materials and Methods

### 4.1. Reagents

Sucrose, Triton X-100, triethylammonium bicarbonate, potassium phosphates, deoxycholic acid sodium salt, urea, 4-vinylpyridine, CNBr-activated Sepharose 4B, and isatin were purchased from Sigma (St. Louis, MO, USA); formic acid was obtained from Merck (Darmstadt, Germany); acetonitrile was obtained from Fisher Chemical (Leicestershire, UK); and tris-(2-carboxyethyl) phosphine was obtained from Pierce-Thermo Scientific (Rockford, IL, USA). Trypsin (modified sequencing grade) was obtained from Promega (Madison, WI, USA), and 5-aminoisatin was synthesized using standard methods [84].

### 4.2. Animals and Isatin Administration

Sixteen male C57BL/6 mice (20–25 g; n = 8 in each group), obtained from the Stolbovaya nursery (Moscow region), were used in this study. Experiments were performed one week after their arrival from the nursery. Animals were kept under natural illumination and received standard laboratory chow and water ad libitum. Isatin was injected subcutaneously (s.c., 100 mg/kg); control mice were treated with the same volume of saline (0.1 mL/kgl s.c.). All procedures conformed to the Russian version of the Guide for the Care and Use of Laboratory Animals (Washington, 1996) were approved by the Animal Care and Use Committee of the Institute of Biomedical Chemistry (approval certificated issued from November 12, 2019, local code IBMC-RFBR-00042).

### 4.3. Sample Preparation for Transcriptome Analysis and Proteomic Profiling of Isatin-Binding Proteins

The animals were decapitated 24 h after injection under light ether anesthesia. The extracted brains were quickly washed in ice-cold saline, and brain hemispheres were immediately used in subsequent experiments. Four samples per group of mice were used for transcriptomic and proteomic analyses.

#### 4.3.1. Transcriptome Analysis

Total RNA from brain hemispheres samples (~15–20 mg) was isolated using the Absolutely RNA Miniprep Kit (Agilent, cat. no. 5190-0444). The quality of obtained RNA samples was assessed using an Agilent Bioanalyser 2100 (Agilent, Santa Clara, CA, USA). RNA samples with an RIN of at least 8 were used for subsequent analysis. Sample preparation was carried out in accordance with the Two Color Microarray-Based Gene Expression Analysis Version 6.6 protocol for two-color hybridization using the two-color Low input Quick-Amp Labeling Kit (cat. no. 5190-2331; Agilent, Santa Clara, CA, USA). The experimental samples were labeled with Cy5 –CTP, and the reference RNA (Universal Mouse Reference RNA (cat. no. 740100; Agilent, Santa Clara, CA, USA) was labeled with Cy3-CTP. According to the standard protocol recommended by the manufacturer, the corresponding amounts of RNA from the Agilent RNA Spike-In Kit (cat. no. 5188-5279; Agilent, Santa Clara, CA, USA) were added to the samples. Evaluation of gene expression was carried out using microarrays for mouse whole-genome microarray analysis (cat. no. G2519F-026655; 4x44K V2 format; Agilent, Santa Clara, CA, USA).

After fragmentation of cRNA and hybridization performed in accordance with the standard protocol, data acquisition was performed by using a DNA Microarray Scanner G2505C (Agilent, Santa Clara, CA, USA).

Primary transcriptome data were treated using Feature Extraction software (version 10.1.3.1; Agilent Technologies).

Further data processing was performed using the GeneXplain platform [85]. Non-logarithmic normalization of the raw data for Control and Isatin treatment conditions was carried out pairwise using module “Normalize Agilent experiment and control” with methods in the Bioconductor LIMMA package. The up- and down-regulated transcripts were identified, and the log-fold change values were calculated by the workflow “Compute differentially expressed genes (Agilent probes)”. This up and down identification analysis applies Student’s *t*-test and calculates *p* values. The following criteria were used: LogFoldChange > 0.5 and –log_*p*_value > 3 for upregulated probes; LogFoldChange < –0.5 and –log_*p*_value < –3 for downregulated probes.

#### 4.3.2. Functional Analysis of Differentially Expressed Genes

Functional analysis of up- and down-regulated genes was performed individually using the GeneXplain platform with default settings of the “Mapping to ontologies (PROTEOME™)” workflow. The “Functional classification” module was used for the annotation of differentially expressed genes in terms of Gene Ontology. The algorithm of the functional analysis is intended to detect statistically significant representation of certain functional groups of genes among all genes of the input sets. Statistical significance of the classification was estimated with the adjusted *p* value, and only statistically significant GO groups with adjusted *p* value < 0.05 were considered.

#### 4.3.3. Affinity Chromatography and Sample Preparation for Mass Spectrometric Analysis

The brain hemispheres were immediately dissected and homogenized using a SilentCrusher S homogenizer (Heidolph, Germany) at 50,000 rpm in 0.05 M potassium phosphate buffer, pH 7.4 (buffer A) (1:3, w/v). Homogenates were then lysed with 3% Triton X-100 (final concentration) at 4 °C for 1 h, diluted three-fold with buffer A, and centrifuged at 16,000 rpm (Eppendorf 5415 R centrifuge; Eppendorf, Germany) for 20 min at 4 °C.

5-aminoisatin-Sepharose was prepared as in [38]. Lysates of brain hemisphere homogenates (protein concentration of about 10 mg/mL) were added to 5-aminoisatin-Sepharose and incubated in a suspension (1:1) at 4 °C overnight under gentle stirring.

The affinity sorbent was washed with 100 volumes of buffer A to remove non-specifically bound proteins, and then the remaining proteins were eluted using a column (1 × 2 cm) at room temperature: initially, with 30 mL of 1 mM isatin in buffer A, then with the same volume of 1 M NaCl in buffer A. The eluate (30 mL) was concentrated to 0.25 mL using an Amicon Ultra centrifuge device (Millipore, Bedford, MA, USA). Proteins were extracted with a mixture of chloroform–methanol [35]; trypsinolysis was performed directly on a Vivaspin 500 centrifuge membrane filter (Sartorius Stedim Biotech, Germany) with a 10,000 Da membrane, as described in [35].

Samples were evaporated using a 5301 vacuum concentrator (Eppendorf, Germany), dissolved in 0.1% formic acid, and analyzed using LC-MS/MS.

In order to determine proteins non-specifically bound to the affinity sorbent, control CNBr-activated Sepharose was used; it was subjected to the same procedures as 5-aminoisatin-Sepharose, but without the addition of the affinity ligand.

#### 4.3.4. Mass Spectrometric Analysis

Chromatographic separation was performed using an Ultimate 3000 RSLC Nano system (Thermo Scientific, Rockford, IL, USA). A sample (2 μL) was loaded for 4 min in mobile phase C (water, 2.5% acetonitrile, 0.1% formic acid, 0.03% acetic acid) onto enrichment column. Peptides were separated in a gradient of mobile phase A (aqueous solution of 0.01% formic acid, 0.03% acetic acid), and mobile phase B (90% acetonitrile, 10% methanol, 0.1% formic acid, 0.03% acetic acid) on an Acclaim Pepmap^®^ analytical column (geometry 75 μm × 150 mm, 1.8 μm; Thermo Scientific, Rockford, IL, USA). Initial conditions for the elution gradient were 98% A: 2% B, flow rate of 0.3 μL/min, and the loading rate on the enrichment column was 15 μL/min. To increase the washing efficiency of the column during chromatographic separation (removal of bound hydrophobic components), the dynamic change in the flow rate to 0.45 mL/min was used at a high relative content of the mobile phase B.

Mass spectrometric analysis was performed on an Orbitrap Fusion high-resolution hybrid mass spectrometer (Thermo Scientific, Rockford, IL, USA) with an NSI ionization source in the mode of positive electrostatic ionization. Precursor ions were acquired at a resolution of 60K in the *m/z* range of 425–1250 with the maximum integration time of 15 ms or a minimum. Precursor ions with charge state z = 2+ … 4+ were isolated with an isolation window of ± 0.75 *m/z* and offset of 0.25 *m/z* and triggered for fragmentation in HCD (higher-energy collision dissociation) mode with the normalized activation energy at 27%. Fragment ions were acquired in an orbital mass analyzer with a resolution of 15K and with a maximum accumulation time of 85 ms.

Data files obtained after LC-MS/MS analysis were converted in peak list format and used for protein identification. Peak lists obtained from spectra were identified using X!Tandem, and the search was conducted using Search GUI version 2.3.17 (Compomics, Belgium) [86]. Proteins were identified against a concatenated target/decoy database of human proteins (Uniprot database revision April 2019). The decoy sequences were created by reversing the target sequences in Search GUI. The identification settings were as follows: trypsin as a specific protease, with a maximum of 1 missed cleavage, tolerance of ±5 ppm as the MS1 level, and tolerance of ±0.005 Da as the MS2 level tolerances; variable modifications: oxidation of M (+15.99), deamination of N (+0.98 u), deamination of Q (+0.98 u); cysteine pyridilethylation C (+105 u) was set as a fixed modification. Peptide Spectrum Matches (PSMs), peptides, and proteins were validated at a 1.0% false discovery rate (FDR) estimated using the decoy hit distribution [87]. Proteins were inferred from the spectrum identification results and quantified using normalized spectrum abundance factor (NSAF) in Peptide Shaker version 1.16.15 (Compomics, Belgium) [88,89].

In each proteomic experiment, the total preparations obtained during the affinity enrichment of lysates of brain homogenates of at least two mice were used. Each of the proteins presented in the tables was identified in at least three independent experiments.

## 5. Conclusions

**Figure 5:**
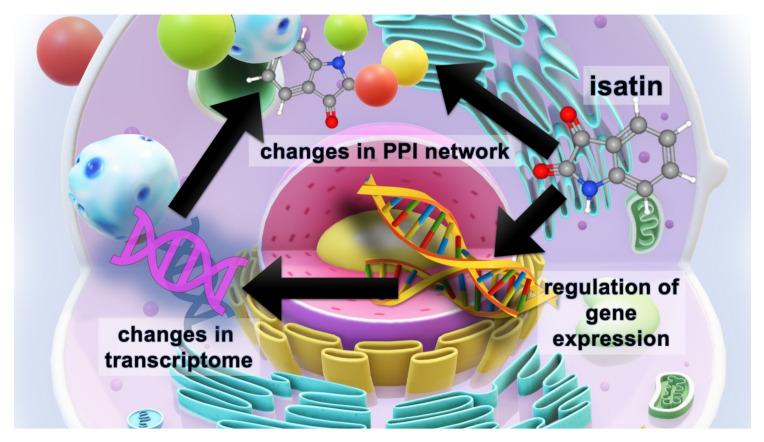
The scheme illustrating the multilevel effects of the neuroprotective dose of isatin (100 mg/kg, 24 h). Isatin has a significant impact on the expression of responsive genes and thus influences the cell transcriptome. Acting on isatin-binding proteins, isatin changes protein–protein interactions (PPI), which are also influenced by the altered proteome, thus causing global changes in the PPI network.

Isatin (indole-2,3-dione) is an endogenous indole found in mammalian tissues and body fluids. It was originally identified as an endogenous MAO (B) inhibitor [90]. Now good evidence exists that it exhibits various biological functions which are unrelated to *MAO* inhibition. Previous studies performed in vitro and in cell cultures revealed a large group of isatin-binding proteins, including nucleoproteins, and also some isatin-responsive genes [1,36,37,38,41,42,43,44,45,46]. This suggests that isatin could be potentially involved in the multilevel regulatory hierarchy from genes to proteins (and their posttranslational modifications) [1]. This study was the first research aimed at investigation of the effect of the neuroprotective dose of isatin to mice (100 mg/kg; 24 h) on gene expression and also on the profile of isatin-binding proteins in the brain hemispheres. Results of transcriptome and proteome analyses of differentially expressed genes suggest the lack of concerted changes 24 h after administration of the neuroprotective dose of isatin. This may be attributed to the known phenomenon of uncoupling of transcription and translation, when some delay between transcription and translation occurs in the course of dynamic adaptation processes [55]. Whether this delay is associated with isatin binding to nucleoproteins [1] and altered ribosome functioning remains to be experimentally investigated. Little correspondence between proteomic and transcriptomic datasets requires the use of both independent validation approaches (e.g., Western blot and qPCR, respectively) and a significant extension of time intervals after isatin administration. Figure 5 summarizes the main results of this study and further research hotspots.

The results of this study have shown that isatin administration produces multilevel changes at least in the brain hemispheres, which include altered gene expression and changes in the profiles of isatin-binding proteins and their interactomes. Appearance of isatin-binding proteins that were not found in the hemispheres of control mice could be associated with changes in PPI networks induced by the accumulation of a high isatin concentration in the brain.

Since isatin-responsive genes (Table 2) and isatin-binding proteins are involved in the control of various important cell functions, it is reasonable to suggest that some isatin analogues may selectively act on particular targets in various brain regions and produce a certain therapeutic effect. The latter is especially important because many isatin-binding proteins have also been implicated in various neurodegenerative processes.

## Figures and Tables

**Table 1:** Differentially expressed proteins detected in brain hemispheres 24 h after administration of a neuroprotective dose of isatin (100 mg/kg, s.c.).

	Entry	Entry Name	ANOVA (*p*)	Fold Change	Description	Function
1	Q9JLV5	*CUL3*	0	−2.681	Cullin-3	E3 ubiquitin ligase of the ubiquitin-proteasome system
2	O35143	*ATIF1*	1.44 × 10^−15^	−2.655	ATPase inhibitor, mitochondrial	Regulation of ATP synthesis
3	P34022	*RANG*	2.00 × 10^−15^	−2.736	Ran-specific GTPase-activating protein	Regulation of nucleocytoplasmic transport
4	Q921I1	*TRFE*	1.11 × 10^−14^	−2.062	Serotransferrin	Binding of iron ions and stimulation of cell proliferation
5	Q8C8N2	*SCAI*	9.71 × 10^−14^	−2.521	Protein SCAI	Tumor suppressor
6	Q9Z1R2	*BAG6*	1.32 × 10^−13^	−2.275	Large proline-rich protein BAG6	BAG family molecular chaperone regulator 6 is involved in DNA damage-induced apoptosis
7	Q61543	*GSLG1*	9.94 × 10^−12^	−2.164	Golgi apparatus protein 1	E-selectin ligand 1, cysteine-rich fibroblast growth factor receptor
8	Q9D2M8	*UB2V2*	1.38 × 10^−11^	−2.224	Ubiquitin-conjugating enzyme E2 variant 2	The component of the ubiquitin-proteasome system
9	P70670	*NACAM*	4.60 × 10^−11^	−2.293	Nascent polypeptide-associated complex subunit alpha, muscle-specific form	Molecular chaperone
10	Q8BGD9	*IF4B*	1.88 × 10^−10^	−2.453	Eukaryotic translation initiation factor 4B	Protein translation
11	Q00623	*APOA1*	4.70 × 10^−10^	−2.363	Apolipoprotein A-I	In CNS, participates in reverse cholesterol transport
12	P84309	*ADCY5*	6.87 × 10^−10^	−2.72	Adenylate cyclase type 5	Cell signaling
13	Q99MN9	*PCCB*	7.83 × 10^−10^	−2.249	Propionyl-CoA carboxylase beta chain, mitochondrial	Fatty acid beta-oxidation
14	P07759	*SPA3K*	4.90 × 10^−9^	−4.156	Serine protease inhibitor A3K	A member of serine protease inhibitors, required for normal synaptic plasticity and regulation of serine protease mediated cell death
15	Q9EPN1	*NBEA*	5.62 × 10^−9^	−3.393	Neurobeachin	A protein required for central synapses formation and functioning
16	P22599	*A1AT2*	5.78 × 10^−9^	−2.595	Alpha-1-antitrypsin 1-2	A member of serine protease inhibitors, required for normal synaptic plasticity and regulation of serine protease-mediated cell death
17	Q62059	*CSPG2*	2.58 × 10^−8^	−2.126	Versican core protein	A component of the extracellular matrix component of the brain
18	D3YXK2	*SAFB1*	3.06 × 10^−8^	−2.383	Scaffold attachment factor B1	Binds to specific sequences of DNA needed for genome organization into functional units in the cell nucleus
19	Q9D1X0	*NOL3*	3.43 × 10^−7^	−3.614	Nucleolar protein 3	An anti-apoptotic protein implicated in down-regulation of activities of caspases-2 and -8 and tumor protein p53
20	P08414	*KCC4*	7.85 × 10^−7^	–10.97	Calcium/calmodulin-dependent protein kinase type IV	Cell signaling
21	Q9D2N4	*DTNA*	1.05 × 10^−6^	−2.133	Dystrobrevin alpha	Required for stability of synapses and clustering of nicotinic acetylcholine receptors
22	P52760	*UK114*	3.24 × 10^−6^	−2.077	Ribonuclease UK114	Endoribonuclease causing translation inhibition by cleaving mRNA
23	Q922D8	*C1TC*	4.95 × 10^−6^	−2.019	C-1-tetrahydrofolate synthase, cytoplasmic	Crucial for de novo synthesis of purines
24	Q8C0E2	*VP26B*	9.11 × 10^−6^	−2.027	Vacuolar protein sorting-associated protein 26B	A component of the retromer cargo-selective complex (CSC)
25	Q6NZL0	*SOGA3*	1.70 × 10^−5^	−2.171	Protein SOGA3	A member of the SOGA (suppressor of glucose by autophagy) family involved in regulation of autophagy
26	Q61029	*LAP2B*	2.69 × 10^−5^	−2.127	Lamina-associated polypeptide 2, isoforms beta/delta/epsilon/gamma	Involved in maintenance of the structural organization of the nuclear envelope and DNA binding
27	O55091	*IMPCT*	2.85 × 10^−5^	−2.608	Protein IMPACT	Translational regulator that helps to maintain constant high levels of translation under stress conditions
28	P35235	*PTN11*	2.89 × 10^−5^	−2,467	Tyrosine-protein phosphatase non-receptor type 11	Participates in the signal transduction from the cell surface to the nucleus
29	P23492	*PNPH*	4.66 × 10^−5^	−2.104	Purine nucleoside phosphorylase	The enzyme involved in purine metabolism
30	Q9QXD6	*F16P1*	1.31 × 10^−4^	−8.295	Fructose-1,6-bisphosphatase 1	Glycolysis and non-canonical functions including blockade of the transcriptional activity of HIF-1α and activation of the RAS/RAF/MEK/ERK cascade
31	O88456	*CPNS1*	6.14 × 10^−3^	−2.523	Calpain small subunit 1	The regulatory subunit of the protease calpain, responsible for limited proteolysis of substrates involved in cytoskeleton remodeling and signal transduction

**Table 2:** Top 20 upregulated genes detected in brain hemispheres 24 h after the administration of the neuroprotective dose of isatin (100 mg/kg, s.c.) to mice.

Gene Symbol	Gene Description	−Log (*p* value)	LogFold Change	Role/Effect
**Upregulated genes**				
*Ctla2a*	cytotoxic T lymphocyte-associated protein 2 alpha	4.154	3.132	Induces apoptosis in some cells
*Nfkbia*	alpha, nuclear factor of kappa light polypeptide gene enhancer in B cells inhibitor	4.191	2.876	Negative regulator of cell proliferation
*Ctla2b*	cytotoxic T lymphocyte-associated protein 2 beta	4.376	2.735	Induces apoptosis in some cells
*Cdkn1a*	cyclin-dependent kinase inhibitor 1A (P21)	3.165	2.730	Delays or stops the cell cycle
*Errfi1*	ERBB receptor feedback inhibitor 1	4.676	2.236	Negative regulator of several EGFR family members
*Sgk1*	serum/glucocorticoid regulated kinase 1	3.642	1.940	Regulation of vascular cell proliferation
*Trp53inp1*	transformation related protein 53 inducible nuclear protein 1	3.301	1.887	One of the key elements in p53-mediated cell cycle arrest and apoptosis in different cell types
*Mtor*	mechanistic target of rapamycin (serine/threonine kinase)	4.091	1.787	Involved in the regulation of cell growth, proliferation, autophagy, and protein synthesis
*Cdt1*	chromatin licensing and DNA replication factor 1	4.554	1.747	DNA replication
*Xpo7*	exportin 7	4.626	1.722	A member of the exportin family, responsible for nucleocytoplasmic trafficking of regulatory proteins
*Gch1*	GTP cyclohydrolase 1	4.055	1.716	Enzyme involved in tetrahydrobiopterin (BH4) biosynthesis; its expression is increased in pathological proliferation
*Epc2*	enhancer of polycomb homolog 2 (Drosophila)	3.578	1.691	Epc plays an important role in cellular differentiation and development
*Xpo4*	exportin 4	4.535	1.608	A member of the exportin family, responsible for nucleocytoplasmic trafficking of regulatory proteins
*Rbbp6*	retinoblastoma binding protein 6	4.721	1.606	Acts as an E3 ubiquitin ligase; is a negative regulator of p53 and promotes cell proliferation
*Ubqln1*	ubiquilin 1	3.187	1.596	Involved in regulation of the protein quality control system
*Recql4*	RecQ protein-like 4	3.030	1.575	DNA helicase that belongs to the RecQ helicase family
*Sap30*	sin3 associated polypeptide 30 kDa	3.040	1.575	Involved in the functional recruitment of the Sin3-histone deacetylase complex
*Stac3*	SH3 and cysteine rich domain 3	4.708	1.573	Involved in neuron specific signal transduction
*Srsf6*	serine/arginine-rich splicing factor 6	3.077	1.457	A member of the conserved family of RNA-binding proteins, which play an important role in the regulation of gene expression
*Pnrc1*	proline-rich nuclear receptor coactivator 1	3.679	1.453	Interacts with various nuclear receptors and functions as a tumor suppressor
**Downregulated genes**				
*Cx3cr1*	chemokine (C-X3-C) receptor 1	−3.709	−2.796	Has a significant impact on cell proliferation
*Tiam2*	T cell lymphoma invasion and metastasis 2	−5.154	−2.105	Plays an important role in neuron development and malignant proliferation in humans
*Slc38a5*	member 5, solute carrier family 38	−3.087	−2.103	Members of this superfamily of solute carriers are crucial for brain physiology and are implicated in various brain disorders
*Lyl1*	lymphoblastomic leukemia 1	−4.446	−1.956	Regulates expression of CREB1 target genes
*Ier5l*	immediate early response 5-like	−3.599	−1.910	Regulates the cellular response to mitogenic signals
*Cyth4*	cytohesin 4	−5.331	−1.882	This guanine nucleotide-exchange protein is an important regulator of signal transduction
*Limd2*	LIM domain containing 2	−3.070	−1.867	Is necessary for growth, development, and adaptive responses
*Gpr17*	G protein-coupled receptor 17	−3.345	−1.853	Cell signaling
*Rasl10a*	RAS-like, family 10, member A	−3.816	−1.760	A Ras-related protein that belongs to the superfamily of small GTPases and exhibits tumor suppressor potential.
*Bcl11a*	B cell CLL/lymphoma 11A (zinc finger protein)	−3.983	−1.759	DNA binding protein
*Tgfb1i1*	transforming growth factor beta 1 induced transcript 1	−5.006	−1.748	A molecular adapter involved in coordination of multiple protein–protein interactions
*Cxcl12*	chemokine (C-X-C motif) ligand 12	−3.073	−1.685	A ligand for CXC chemokine receptor 4 involved in neuronal regeneration in the adult brain
*Cckbr*	cholecystokinin B receptor	−3.012	−1.683	In the brain it is crucial for cortical development
*Tm4sf1*	transmembrane 4 superfamily member 1	−3.198	−1.681	A cell surface glycoprotein involved in cell–cell interactions
*Tbcc*	tubulin-specific chaperone C	−5.848	−1.629	Involved in structural rearrangements of tubulin and actin during the cell cycle
*Aif1*	allograft inflammatory factor 1	−3.263	−1.627	A calcium-binding adapter molecule specifically expressed in microglia.
*Trim59*	tripartite motif-containing 59	−4.371	−1.602	Functions as a ubiquitination ligase; its overexpression stimulates cell proliferation and migration
*Pbx2*	pre B cell leukemia homeobox 2	−5.499	−1.579	A transcription factor required for normal brain development
*Kif21b*	kinesin family member 21B	−4.166	−1.497	An ATP-dependent microtubule-based motor protein involved in the intracellular transport processes
*Rapgef3*	Rap guanine nucleotide exchange factor (GEF) 3	−3.312	−1.488	Involved in brain development and brain diseases

## Supplementary Materials

Supplementary materials can be found at https://www.mdpi.com/1422-0067/21/11/4187/s1.

## References

[1] Medvedev A., Buneeva O., Gnedenko O., Ershov P., Ivanov A. (2018). Isatin, an endogenous non-peptide biofactor: A review of its molecular targets, mechanisms of actions and their biomedical implications. Biofactors.

[2] Phogat P., Singh P. (2015). A mini review on central nervous system potential of isatin derivatives. Cent. Nerv. Syst. Agents Med. Chem..

[3] Medvedev A., Buneeva O., Glover V. (2007). Biological targets for isatin and its analogues: Implications for therapy. Biologics.

[4] Medvedev A., Igosheva N., Crumeyrolle-Arias M., Glover V. (2005). Isatin: Role in stress and anxiety. Stress.

[5] Medvedev A.E., Clow A., Sandler M., Glover V. (1996). Isatin—A link between natriuretic peptides and monoamines?. Biochem. Pharmacol..

[6] Chatterjee S., Lee L.Y., Kawahara R., Abrahams J.L., Adamczyk M.A., Anugraham M., Ashwood C., Sumer-Bayraktar Z., Briggs M.T., Chik J.H.L. (2010). Protein Paucimannosylation Is an Enriched N ‐Glycosylation Signature of Human Cancers. Proteomics.

[7] Pandeya S.N., Smitha S., Jyoti M., Sridhar S.K. (2005). Biological activities of isatin and its derivatives. Acta Pharm..

[8] Vine K.L., Matesic L., Locke J.M., Ranson M., Skropeta D. (2009). Cytotoxic and anticancer activities of isatin and its derivatives: A comprehensive review from 2000–2008. Anticancer Agents Med. Chem..

[9] Pakravan P., Kashanian S., Khodaei M.M., Harding F.J. (2013). Biochemical and pharmacological characterization of isatin and its derivatives: From structure to activity. Pharmacol. Rep..

[10] Verma M., Pandeya S.N., Singh K.N., Stables J.P. (2004). Anticonvulsant activity of Schiff bases of isatin derivatives. Acta Pharm..

[11] Chohan Z.H., Pervez H., Rauf A., Khan K.M., Supuran C.T. (2004). Isatin-derived antibacterial and antifungal compounds and their transition metal complexes. J. Enzyme Inhib. Med. Chem..

[12] Sriram D., Bal T.R., Yogeeswari P. (2004). Design, synthesis and biological evaluation of novel non-nucleoside HIV-1 reverse transcriptase inhibitors with broad-spectrum chemotherapeutic properties. Bioorg. Med. Chem..

[13] Mak N.K., Leung C.Y., Wei X.Y., Shen X.L., Wong R.N., Leung K.N., Fung M.C. (2004). Inhibition of RANTES expression by indirubin in influenza virus-infected human bronchial epithelial cells. Biochem. Pharmacol..

[14] Selvam P., Murgesh N., Chandramohan M., De Clercq E., Keyaerts E., Vijgen L., Maes P., Neyts J., Ranst M.V. (2008). In vitro antiviral activity of some novel isatin derivatives against HCV and SARS-CoV viruses. Indian J. Pharm. Sci..

[15] Chapman J.G., Magee W.P., Stukenbrok H.A., Beckius G.E., Milici A.J., Tracey W.R. (2002). A novel nonpeptidic caspase-3/7 inhibitor, (S)-(+)-5-[1-(2-methoxymethylpyrrolidinyl)sulfonyl]isatin reduces myocardial ischemic injury. Eur. J. Pharmacol..

[16] Limpachayaporn P., Schäfers M., Haufe G. (2015). Isatin sulfonamides: Potent caspases-3 and -7 inhibitors, and promising pet and spect radiotracers for apoptosis imaging. Future Med. Chem..

[17] Motzer R.J., Escudier B., Gannon A., Figlin R.A. (2017). Sunitinib: Ten Years of Successful Clinical Use and Study in Advanced Renal Cell Carcinoma. Oncologist.

[18] Watkins P., Clow A., Glover V., Halket J., Przyborowska A., Sandler M. (1990). Isatin, regional distribution in rat brain and tissues. Neurochem. Int..

[19] Manabe S., Gao Q., Yuan J., Takahashi T., Ueki A. (1997). Determination of isatin in urine and plasma by high-performance liquid chromatography. J. Chromatogr. B Biomed. Sci. Appl..

[20] Mawatari K., Segawa M., Masatsuka R., Hanawa Y., Iinuma F., Watanabe M. (2001). Fluorimetric determination of isatin in human urine and serum by liquid chromatography postcolumn photoirradiation. Analyst.

[21] Igosheva N., Matta S., Glover V. (2004). Effect of acute stress and gender on isatin in rat tissues and serum. Physiol. Behav..

[22] Tozawa Y., Ueki A., Manabe S., Matsushima K. (1998). Stress-induced increase in urinary isatin excretion in rats: Reversal by both dexamethasone and alpha-methyl-P-tyrosine. Biochem. Pharmacol..

[23] Bhattacharya S.K., Clow A., Przyborowska A., Halket J., Glover V., Sandler M. (1991). Effect of aromatic amino acids, pentylenetetrazole and yohimbine on isatin and tribulin activity in rat brain. Neurosci. Lett..

[24] Sandler M., Przyborowska A., Halket J., Watkins P., Glover V., Coates M.E. (1991). Urinary but not brain isatin levels are reduced in germ-free rats. J. Neurochem..

[25] Bhattacharya S.K., Mitra S.K., Acharya S.B. (1991). Anxiogenic activity of isatin, a putative biological factor, in rodents. J. Psychopharmacol..

[26] Bhattacharya S.K., Acharya S.B. (1993). Further investigations on the anxiogenic effects of isatin. Biog. Amines.

[27] Abel E.L. (1995). Behavioral effects of isatin on open field activity and immobility in the forced swim test in rats. Physiol. Behav..

[28] Chocholova L., Kolinova M. (1979). Effect of isatin (2,3-Dioxoindoline) on audiogenic seizures in rats and its relationship to electragraphic and behavioural phenomena. Physiol. Bohemoslov..

[29] Medvedev A.E., Gorkin V.Z., Fedotova I.B., Semiokhina A.F., Sandler M., Glover V. (1994). Antiseizure effect of isatin and reduction of monoamine oxidase activity in rats with experimental audiogenic seizures. Med. Sci. Res..

[30] Bhattacharya S.K., Charkrabarti A. (1998). Dose-related proconvulsant and anticonvulsant activity of isatin, a putative biological factor in rats. Ind. J. Exp. Biol..

[31] Zhou Y., Zhao Z.Q., Xie J.X. (2001). Effect of isatin on rotational behavior and DA levels in caudate putamen in Parkinsonian rats. Brain Res..

[32] Hamaue N., Minami M., Terado M., Hirafuji M., Endo T., Machida M., Hiroshige T., Ogata A., Tashiro K., Saito H. (2004). Comparative study of the effects of isatin, an endogenous MAO-inhibitor, and selegiline on bradykinesia and dopamine levels in a rat model of Parkinson’s disease induced by the Japanese encephalitis virus. Neurotoxicology.

[33] Minami M., Hamaue N., Hirafuji M., Saito H., Hiroshige T., Ogata A., Tashiro K., Parvez S.H. (2006). Isatin, an endogenous MAO inhibitor, and a rat model of Parkinson’s disease induced by the Japanese encephalitis virus. J. Neural Transm..

[34] Medvedev A.E., Buneeva O.A., Kopylov A.T., Tikhonova O.V., Medvedeva M.V., Nerobkova L.N., Kapitsa I.G., Zgoda V.G. (2017). Brain mitochondrial subproteome of Rpn10-binding proteins and its changes induced by the neurotoxin MPTP and the neuroprotector isatin. Biochemistry.

[35] Buneeva O., Kopylov A., Kapitsa I., Ivanova E., Zgoda V., Medvedev A. (2018). The effect of neurotoxin MPTP and neuroprotector isatin on the profile of ubiquitinated brain mitochondrial proteins. Cells.

[36] Crumeyrolle-Arias M., Buneeva O., Zgoda V., Kopylov A., Cardona A., Tournaire M.-C., Pozdnev V., Glover V., Medvedev A. (2009). Isatin binding proteins in rat brain: In situ imaging, quantitative characterization of specific [^3^H]isatin binding, and proteomic profiling. J. Neurosci. Res..

[37] Buneeva O., Gnedenko O., Zgoda V., Kopylov A., Glover V., Ivanov A., Medvedev A., Archakov A. (2010). Isatin binding proteins of rat and mouse brain: Proteomic identification and optical biosensor validation. Proteomics.

[38] Buneeva O.A., Kopylov A.T., Tikhonova O.V., Zgoda V.G., Medvedev A.E., Archakov A.I. (2012). Effect of affinity sorbent on proteomic profiling of isatin-binding proteins of mouse brain. Biochemistry.

[39] Medvedev A.E., Buneeva O.A., Kopylov A.T., Gnedenko O.V., Medvedeva M.V., Kozin S.A., Ivanov A.S., Zgoda V.G., Makarov A.A. (2015). The effects of an endogenous non-peptide molecule isatin and hydrogen peroxide on proteomic profiling of rat brain amyloid-beta binding proteins: Relevance to Alzheimer’s disease?. Int. J. Mol. Sci..

[40] Medvedev A., Buneeva O., Gnedenko O., Fedchenko V., Medvedeva M., Ivanov Y., Glover V., Sandler M. (2006). Isatin interaction with glyceraldehyde-3-phosphate dehydrogenase, a putative target of neuroprotective drugs: Partial agonism with deprenyl. J. Neural Transm..

[41] Cane A., Tournaire M.C., Barritault D., Crumeyrolle-Arias M. (2000). The endogenous oxindoles 5-hydroxyoxindole and isatin are antiproliferative and proapoptotic. Biochem. Biophys. Res. Commun..

[42] Igosheva N., Lorz C., O’Conner E., Glover V., Mehmet H. (2005). Isatin, an endogenous monoamine oxidase inhibitor, triggers a dose- and time-dependent switch from apoptosis to necrosis in human neuroblastoma cells. Neurochem. Int..

[43] Hou L., Ju C., Zhang J., Song J., Ge Y., Yue W. (2008). Antitumor effects of isatin on human neuroblastoma cell line (SH-SY5Y) and the related mechanism. Eur. J. Pharmacol..

[44] Song J., Hou L., Ju C., Zhang J., Ge Y., Yue W. (2013). Isatin inhibits proliferation and induces apoptosis of SH-SY5Y neuroblastoma cells in vitro and *in vivo*. Eur. J. Pharmacol..

[45] Ma Z., Hou L., Jiang Y., Chen Y., Song J. (2014). The endogenous oxindole isatin induces apoptosis of MCF 7 breast cancer cells through a mitochondrial pathway. Oncol. Rep..

[46] Sun W., Zhang L., Hou L., Ju C., Zhao S., Wei Y. (2017). Isatin inhibits SH-SY5Y neuroblastoma cell invasion and metastasis through MAO/HIF-1α/CXCR4 signaling. Anticancer Drugs.

[47] Fogaça M.V., Cândido-Bacani P.M., Benicio L.M., Zapata L.M., Cardoso P.F., de Oliveira M.T., Calvo T.R., Varanda E.A., Vilegas W., de Syllos Colus I.M. (2017). Effects of indirubin and isatin on cell viability, mutagenicity, genotoxicity and BAX/ERCC1 gene expression. Pharm. Biol..

[48] Buneeva O.A., Kapitsa I.G., Ivanova E.A., Kopylov A.T., Zgoda V.G., Medvedev A.E. (2019). The effect of a neuroprotective dose of isatin or deprenyl to mice on the profile of brain isatin-binding proteins. Biomed. Khim..

[49] Panova N.G., Zemskova M.A., Axenova L.N., Medvedev A.E. (1997). Does isatin interact with rat brain monoamine oxidases *in vivo*?. Neurosci. Lett..

[50] Xu P., Hou L., Ju C., Zhang Z., Sun W., Zhang L., Song J., Lv Y., Liu L., Chen Z. (2016). Isatin inhibits the proliferation and invasion of SHSY5Y neuroblastoma cells. Mol. Med. Rep..

[51] Zhang L., Sun W., Cao Y., Hou L., Ju C., Wang X. (2019). Isatin inhibits the invasion of human SHSY5Y neuroblastoma cells based on microarray analysis. Mol. Med. Rep..

[52] Fedchenko V., Globa A., Kaloshin A., Kapitsa I., Nerobkova L., Val’dman E., Buneeva O., Glover V., Medvedev A. (2008). The effect of short-term administration of (-)-deprenyl and isatin on expression of some genes in the mouse brain cortex. Med. Sci. Monit..

[53] Rehni A.K., Singh T.G., Jaggi A.S., Singh N. (2008). Pharmacological preconditioning of the brain: A possible interplay between opioid and calcitonin gene related peptide transduction systems. Pharmacol. Rep..

[54] Snaebjornsson M.T., Schulze A. (2018). Non-canonical functions of enzymes facilitate cross-talk between cell metabolic and regulatory pathways. Exp. Mol. Med..

[55] Liu Y., Beyer A., Aebersold R. (2016). On the Dependency of Cellular Protein Levels on mRNA Abundance. Cell.

[56] Walch L. (2013). Emerging Role of the Scaffolding Protein Dlg1 in Vesicle Trafficking. Traffic.

[57] Von Ossowski L., Li L.-L., MoÈykkynen T., Coleman S.K., Courtney M.J., KeinaÈnen K. (2017). Cysteine 893 is a target of regulatory thiol modifications of GluA1 AMPA receptors. PLoS ONE.

[58] Saura M., Zaragoza C., Herranz B., Griera M., Diez-Marqués L., Rodriguez-Puyol D., Rodriguez-Puyol M. (2005). Nitric oxide regulates transforming growth factor-beta signaling in endothelial cells. Circ. Res..

[59] Denis J.-F., Sader F., Gatien S., Villiard É., Philip A., Roy S. (2016). Activation of Smad2 but not Smad3 is required to mediate TGF-β signaling during axolotl limb regeneration. Development.

[60] Viswanatha R., Wayt J., Ohouo P.Y., Smolka M.B., Bretscher A. (2013). Interactome analysis reveals ezrin can adopt multiple conformational states. J. Biol. Chem..

[61] McClatchey A.I. (2014). ERM proteins at a glance. J. Cell Sci..

[62] Poullet P., Gautreau A., Kadare G., Girault J.-A., Louvard D., Arpin M. (2001). Ezrin interacts with focal adhesion kinase and induces its activation independently of cell-matrix adhesion. J. Biol. Chem..

[63] Tapial Martínez P., López Navajas P., Lietha D. (2020). FAK structure and regulation by membrane interactions and force in focal adhesions. Biomolecules.

[64] Baillat G., Siret C., Delamarre E., Luis J. (2008). Early adhesion induces interaction of FAK and Fyn in lipid domains and activates raft-dependent Akt signaling in SW480 colon cancer cells. Biochim. Biophys. Acta.

[65] Liu S., Premont R.T., Rockey D. (2012). G-protein-coupled receptor kinase interactor-1 (GIT1) is a new endothelial nitric-oxide synthase (eNOS) interactor with functional effects on vascular homeostasis. J. Biol. Chem..

[66] Zhang H., Webb D.J., Asmussen H., Horwitz A.F. (2003). Synapse formation is regulated by the signaling adaptor GIT1. J. Cell Biol..

[67] Beurel E., Grieco S.F., Jope R.S. (2015). Glycogen synthase kinase-3 (GSK3): Regulation, actions, and diseases. Pharmacol. Ther..

[68] Pilot-Storck F., Chopin E., Rual J.-F., Baudot A., Dobrokhotov P., Robinson-Rechavi M., Brun C., Cusick M.E., Hill D.E., Schaeffer L. (2010). Interactome mapping of the phosphatidylinositol 3-kinase-mammalian target of rapamycin pathway identifies deformed epidermal autoregulatory factor-1 as a new glycogen synthase kinase-3 interactor. Mol. Cell. Proteom..

[69] Popkie A.P., Zeidner L.C., Albrecht A.M., D’Ippolito A., Eckardt S., Newsom D.E., Groden J., Doble B.W., Aronow B., McLaughlin K.J. (2010). Phosphatidylinositol 3-kinase (PI3K) signaling via glycogen synthase kinase-3 (GSK-3) regulates DNA methylation of imprinted loci. J. Biol. Chem..

[70] Karamanou S., Bariami V., Papanikou E., Kalodimos C.G., Economou A. (2008). Assembly of the translocase motor onto the preprotein-conducting channel. Mol. Microbiol..

[71] Rondeau P., Bourdon E. (2011). The glycation of albumin: Structural and functional impacts. Biochimie.

[72] Stoll P., Bassler N., Hagemeyer C.E., Eisenhardt S.U., Chen Y.C., Schmidt R., Schwarz M., Ahrens I., Katagiri Y., Pannen B. (2007). Targeting ligand-induced binding sites on GPIIb/IIIa via single-chain antibody allows effective anticoagulation without bleeding time prolongation. Arterioscler. Thromb. Vasc. Biol..

[73] Ivanov A., Medvedev A., Ershov P., Molnar A., Mezentsev Y., Yablokov E., Kaluzhsky L., Gnedenko O., Buneeva O., Haidukevich I. (2014). Protein interactomics based on direct molecular fishing on paramagnetic particles: Practical realization and further SPR validation. Proteomics.

[74] Ershov P., Mezentsev Y., Gilep A., Usanov S., Buneeva O., Medvedev A., Ivanov A. (2017). Isatin-induced increase in the affinity of human ferrochelatase and adrenodoxin reductase interaction. Protein Sci..

[75] Roberts J.M., Ennajdaoui H., Edmondson C., Wirth B., Sanford J., Chen B. (2014). Splicing factor TRA2B is required for neural progenitor survival. J. Comp. Neurol..

[76] Liu J., Bian T., Feng J., Qian L., Zhang J., Jiang D., Zhang Q., Li X., Liu Y., Shi J. (2018). miR-335 inhibited cell proliferation of lung cancer cells by target Tra2b. Cancer Sci..

[77] Liu N., Zhou K.I., Parisien M., Dai Q., Diatchenko L., Pan T. (2017). N^6^-methyladenosine alters RNA structure to regulate binding of a low-complexity protein. Nucleic Acids Res..

[78] Correa B.R., de Araujo P.R., Qiao M., Burns S.C., Chen C., Schlegel R., Agarwal S., Galante P.A.F., Penalva L.O.F. (2016). Functional genomics analyses of RNA-binding proteins reveal the splicing regulator SNRPB as an oncogenic candidate in glioblastoma. Genome Biol..

[79] Motley W.W., Talbot K., Fischbeck K.H. (2010). GARS axonopathy: Not every neuron’s cup of tRNA. Trends Neurosci..

[80] Yao P., Fox P.L. (2013). 2013 Aminoacyl-tRNA synthetases in medicine and disease. EMBO Mol. Med..

[81] Hou Y., Shang C., Wang H., Yun J. (2020). Isatin-azole hybrids and their anticancer activities. Arch. Pharm..

[82] Gupta A.K., Tulsyan S., Bharadwaj M., Mehrota R. (2019). Systematic review on cytotoxic and anticancer potential of n-substituted isatins as novel class of compounds useful in multidrug-resistant cancer therapy: In silico and in vitro analysis. Top. Curr. Chem..

[83] El-Naggar M., Eldehna W.M., Almahli H., Elgez A., Fares M., Elaasser M.M., Abdel-Aziz H.A. (2018). Novel thiazolidinone/thiazolo[3,2-*a*]benzimidazolone-isatin conjugates as apoptotic anti-proliferative agents towards breast cancer: One-pot synthesis and in vitro biological evaluation. Molecules.

[84] Medvedev A.E., Goodwin B.L., Sandler M., Glover V. (1999). Efficacy of isatin analogues as antagonists of rat brain and heart atrial natriuretic peptide receptors coupled to particulate guanylyl cyclase. Biochem. Pharmacol..

[85] GeneXplain platform, release 3.0. http://platform.genexplain.com/.

[86] Vaudel M., Barsnes H., Berven F.S., Sickmann A., Martens L. (2011). SearchGUI: An open-source graphical user interface for simultaneous OMSSA and X!Tandem searches. Proteomics.

[87] Elias J.E., Gygi S.P. (2007). Target-decoy search strategy for increased confidence in large-scale protein identifications by mass spectrometry. Nat. Methods.

[88] Vaudel M., Burkhart J.M., Zahedi R.P., Oveland E., Berven F.S., Sickmann A., Martens L., Barsnes H. (2015). PeptideShaker enables reanalysis of MS-derived proteomics data sets. Nat. Biotechnol..

[89] McIlwain S., Mathews M., Bereman M.S., Rubel E.W., MacCoss M.J., Noble W.S. (2012). Estimating relative abundances of proteins from shotgun proteomics data. BMC Bioinform..

[90] Glover V., Halket J.M., Watkins P.J., Clow A., Goodwin B.L., Sandler M. (1988). Isatin: Identity with the purified endogenous monoamine oxidase inhibitor tribulin. J. Neurochem..

