# Supplementary material for: A Neuroprotective Dose of Isatin Causes Multilevel Changes Involving the Brain Proteome: Prospects for Further Research
Source: Int J Mol Sci. 2020 Jun 11;21(11):4187. doi: 10.3390/ijms21114187 (PMC7313464; doi:10.3390/ijms21114187)
Supplement: Supplementary file 1 [file ijms-21-04187-s001.zip › manuscript_supplementary/README_Supplements_Structure.rtf]

S01_Downregulated Ensembl genes GO	•Downregulated genes GO BP 	•Cellular compartments	•Molecular  functions	•Biological Processes, level 5	•Biological Processes, level 4S01_Downregulated Ensembl genes	•Downregulated Ensembl genesS01_Downregulated PROTEOME (BP)	•Downregulated PROTEOME (BP)	•DiagramsS01_UpDownReg Ensembl genes GO	•Up and Down Regulated genes GO Biological Processes (BP)	•Biological Processes, level 4	•Biological Processes, level 5	•Cellular compartments	•Molecular  functionsS01_Upregulated Ensembl genes	•Upregulated Ensembl genesS02_Isatin_binding_proteins_all	•Control	•Isatin	•Symmetry	•Isatin specific	•Control specificS03_Control_specific-isatin_binding_proteins	•Control	•Annotation refinedS03_Isatin_specific_isatin_binding_proteins_	•Isatin	•Annotation refined
